# 2-Amino-5-bromo­pyridinium 4-carb­oxy­butano­ate

**DOI:** 10.1107/S1600536810026280

**Published:** 2010-07-10

**Authors:** Madhukar Hemamalini, Hoong-Kun Fun

**Affiliations:** a X-ray Crystallography Unit, School of Physics, Universiti Sains Malaysia, 11800 USM, Penang, Malaysia

## Abstract

In the title salt, C_5_H_6_BrN_2_^+^·C_5_H_7_O_4_^−^, the 2-amino-5-bromo­pyridinium cation is essentially planar, with a maximum deviation of 0.005 (3) Å. In the crystal structure, the proton­ated N atom and the 2-amino group of the cation are hydrogen bonded to the carboxyl­ate O atoms of the anion *via* a pair of N—H⋯O hydrogen bonds, forming an *R*_2_^2^(8) ring motif. The ion pairs are further connected *via* O—H⋯O, N—H⋯O and C—H⋯O hydrogen bonds, forming a two-dimensional network parallel to the *bc* plane. In the network, the hydrogen glutarate (4-carb­oxy­butano­ate) anions self-assemble through O—H⋯O hydrogen bonds, forming a supra­molecular chain along the *c* axis.

## Related literature

For applications of weak intermolecular inter­actions, see: Moghimi *et al.* (2002 ▶); Aghabozorg *et al.* (2005 ▶); Lehn (1992 ▶). For the conformation of glutaric acid, see: Saraswathi *et al.* (2001 ▶). For hydrogen-bond motifs, see: Bernstein *et al.* (1995 ▶). For bond-length data, see: Allen *et al.* (1987 ▶). 
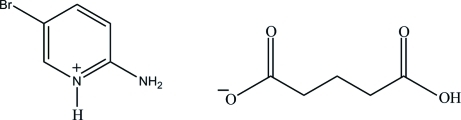


## Experimental

### 

#### Crystal data

C_5_H_6_BrN_2_^+^·C_5_H_7_O_4_^−^
*M* *_r_* = 305.13
Orthorhombic, 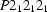

*a* = 5.1499 (12) Å
*b* = 14.858 (4) Å
*c* = 16.022 (4) Å
*V* = 1226.0 (5) Å^3^
*Z* = 4
Mo *K*α radiation
μ = 3.36 mm^−1^
*T* = 296 K
0.72 × 0.31 × 0.15 mm

#### Data collection

Bruker APEXII DUO CCD area-detector diffractometer
Absorption correction: multi-scan (*SADABS*; Bruker, 2009 ▶) *T*_min_ = 0.195, *T*_max_ = 0.628
8937 measured reflections
4149 independent reflections
2911 reflections with *I* > 2σ(*I*)
*R*_int_ = 0.035

#### Refinement

*R*[*F* ^2^ > 2σ(*F* ^2^)] = 0.041
*wR*(*F* ^2^) = 0.116
*S* = 1.02
4149 reflections
170 parameters
H atoms treated by a mixture of independent and constrained refinement
Δρ_max_ = 0.59 e Å^−3^
Δρ_min_ = −0.42 e Å^−3^
Absolute structure: Flack (1983 ▶), 1734 Friedel pairs
Flack parameter: 0.024 (9)

### 

Data collection: *APEX2* (Bruker, 2009 ▶); cell refinement: *SAINT* (Bruker, 2009 ▶); data reduction: *SAINT*; program(s) used to solve structure: *SHELXTL* (Sheldrick, 2008 ▶); program(s) used to refine structure: *SHELXTL*; molecular graphics: *SHELXTL*; software used to prepare material for publication: *SHELXTL* and *PLATON* (Spek, 2009 ▶).

## Supplementary Material

Crystal structure: contains datablocks global, I. DOI: 10.1107/S1600536810026280/is2573sup1.cif

Structure factors: contains datablocks I. DOI: 10.1107/S1600536810026280/is2573Isup2.hkl

Additional supplementary materials:  crystallographic information; 3D view; checkCIF report

## Figures and Tables

**Table 1:** Hydrogen-bond geometry (Å, °)

*D*—H⋯*A*	*D*—H	H⋯*A*	*D*⋯*A*	*D*—H⋯*A*
N1—H1*N*1⋯O2^i^	0.94 (3)	1.73 (3)	2.666 (3)	177 (2)
N2—H1*N*2⋯O1^i^	0.83 (4)	1.99 (4)	2.806 (4)	170 (4)
N2—H2*N*2⋯O1^ii^	0.83 (3)	2.04 (3)	2.848 (3)	164 (3)
O4—H1*O*4⋯O2^iii^	0.69 (5)	1.93 (5)	2.601 (3)	166 (5)
C3—H3*A*⋯O3^iv^	0.93	2.57	3.382 (4)	146
C6—H6*A*⋯O3^v^	0.93	2.46	3.337 (4)	157

Symmetry codes: (i) 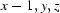; (ii) 
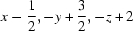
; (iii) 
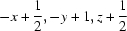
; (iv) 
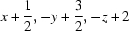
; (v) 
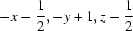
.

**Fig. 1.**
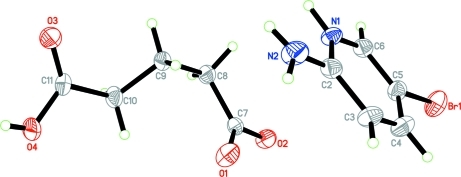
The asymmetric unit of the title compound. Displacement ellipsoids are drawn at the 30% probability level.

**Fig. 2.**
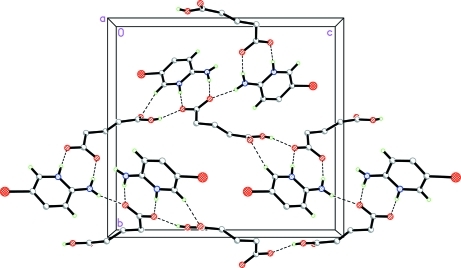
The crystal packing of (I), showing hydrogen-bonded (dashed lines) 2D networks parallel to to the bc-plane. H atoms not involved in the intermolecular interactions have been omitted for clarity.

**Fig. 3.**
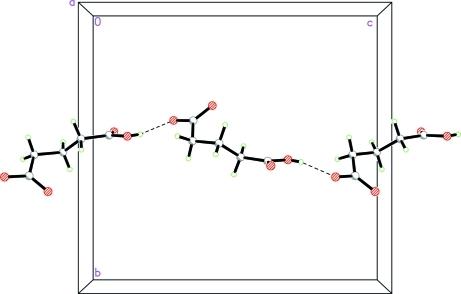
Carboxyl-carboxylate interactions made up of hydrogen glutarate anion.

**Fig. 4.**
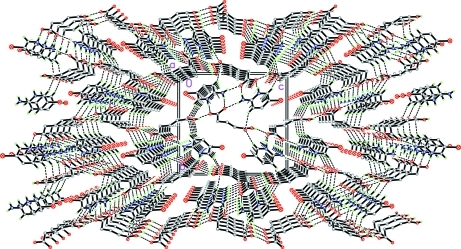
The crystal packing of the title compound (I), showing the stacking of the molecules down the a-axis. H atoms not involved in the intermolecular interactions have been omitted for clarity.

## supplementary crystallographic information

### Comment

Weak interactions, such as hydrogen bonding and π–π stacking, have attracted much interest as a result of their significance in chemistry and biology, especially in the field of crystal engineering (Moghimi *et al.*, 2002; Aghabozorg *et al.*, 2005).  The design of highly specific solid-state compounds is of considerable significance in organic chemistry due to the important applications of these compounds in the development of new optical, magnetic and electronic systems (Lehn, 1992). The present work is part of a structural study of complexes of 2-amino pyridinium systems with hydrogen-bond donors and we report here the structure of 2-amino-5-bromopyridinium hydrogen glutarate, (I).

The asymmetric unit (Fig. 1) contains a 2-amino-5-bromopyridinium cation and a hydrogen glutarate anion. The 2-amino-5-bromopyridinium cation is essentially planar, with a maximum deviation of 0.005 (3) Å for atom C5.  In the 2-amino-5-bromopyridinium cation, a wider than normal angle [C6—N1—C2 = 123.8 (2)°] is subtented at the protonated N1 atom. The backbone  conformation of the hydrogen glutarate anion can be described by the two torsion angles C8-C9-C10-C11 of -178.0 (2)° and C7-C8-C9-C10 of -71.7 (3)°. As evident from the torsion angles, the backbone is in a fully extended conformation (Saraswathi *et al.*, 2001) of the two carboxyl groups, one is deprotonated while the other is not. The bond lengths (Allen *et al.*, 1987) and angles are within normal ranges.

In the crystal packing, the protonated N1 atom and the 2-amino group (N2) is hydrogen-bonded to the carboxylate oxygen atoms (O1 and O2) via a pair of intermolecular N1—H1N1···O2 and N2–H1N2···O1 hydrogen bonds forming a ring motif *R*_2_^2^(8) (Bernstein *et al.*, 1995).  The ion pairs are further connected via N2—H2N2···O1, O4—H1O4···O2 and C6—H6A···O3 (Table 1) hydrogen bonds, forming a two-dimensional network parallel to the *bc*-plane (Fig. 2). The hydrogen glutarate anions self-assemble through O4—H1O4···O2 hydrogen bonds, forming one-dimensional supramolecular chains along the *c*-axis (Fig. 3). Furthermore, the ion pairs are stacked down along the *a*-axis, forming a three-dimensional network as shown in Fig. 4.

### Experimental

A hot methanol solution (20 ml) of 2-amino-5-bromopyridine (86 mg, Aldrich) and glutaric acid (66 mg, Merck) were mixed and warmed over a heating magnetic stirrer hotplate for a few minutes. The resulting solution was allowed to cool slowly at room temperature and crystals of the title compound appeared after a few days.

### Refinement

Atoms H1N1, H1N2, H2N2 and  H1O4 were located from a difference Fourier map and were refined freely [N—H= 0.83 (4)–0.94 (3) Å and O—H = 0.69 (5) Å]. The remaining hydrogen atoms were positioned geometrically [C—H = 0.93 or 0.97 Å] and were refined using a riding model, with *U*_iso_(H) = 1.2*U*_eq_(C). 1734 Friedel pairs were used to determine the absolute configuration.

### Crystal data

**Table:**

C_5_H_6_BrN_2_^+^·C_5_H_7_O_4_^−^	*F*(000) = 616
*M**_r_* = 305.13	*D*_x_ = 1.653 Mg m^−^^3^
Orthorhombic, *P*2_1_2_1_2_1_	Mo *K*α radiation, λ = 0.71073 Å
Hall symbol: P 2ac 2ab	Cell parameters from 2942 reflections
*a* = 5.1499 (12) Å	θ = 2.7–26.8°
*b* = 14.858 (4) Å	µ = 3.36 mm^−^^1^
*c* = 16.022 (4) Å	*T* = 296 K
*V* = 1226.0 (5) Å^3^	Block, colourless
*Z* = 4	0.72 × 0.31 × 0.15 mm

### Data collection

**Table:**

Bruker APEXII DUO CCD area-detector diffractometer	4149 independent reflections
Radiation source: fine-focus sealed tube	2911 reflections with *I* > 2σ(*I*)
graphite	*R*_int_ = 0.035
φ and ω scans	θ_max_ = 31.8°, θ_min_ = 2.7°
Absorption correction: multi-scan (*SADABS*; Bruker, 2009)	*h* = −7→7
*T*_min_ = 0.195, *T*_max_ = 0.628	*k* = −22→21
8937 measured reflections	*l* = −21→23

### Refinement

**Table:**

Refinement on *F*^2^	Secondary atom site location: difference Fourier map
Least-squares matrix: full	Hydrogen site location: inferred from neighbouring sites
*R*[*F*^2^ > 2σ(*F*^2^)] = 0.041	H atoms treated by a mixture of independent and constrained refinement
*wR*(*F*^2^) = 0.116	*w* = 1/[σ^2^(*F*_o_^2^) + (0.0414*P*)^2^] where *P* = (*F*_o_^2^ + 2*F*_c_^2^)/3
*S* = 1.02	(Δ/σ)_max_ = 0.001
4149 reflections	Δρ_max_ = 0.59 e Å^−^^3^
170 parameters	Δρ_min_ = −0.42 e Å^−^^3^
0 restraints	Absolute structure: Flack (1983), 1734 Friedel pairs
Primary atom site location: structure-invariant direct methods	Flack parameter: 0.024 (9)

### Special details

**Table:**

Geometry. All s.u.'s (except the s.u. in the dihedral angle between two l.s. planes) are estimated using the full covariance matrix. The cell s.u.'s are taken into account individually in the estimation of s.u.'s in distances, angles and torsion angles; correlations between s.u.'s in cell parameters are only used when they are defined by crystal symmetry. An approximate (isotropic) treatment of cell s.u.'s is used for estimating s.u.'s involving l.s. planes.
Refinement. Refinement of F^2^ against ALL reflections. The weighted R-factor wR and goodness of fit S are based on F^2^, conventional R-factors R are based on F, with F set to zero for negative F^2^. The threshold expression of F^2^ > 2σ(F^2^) is used only for calculating R-factors(gt) etc. and is not relevant to the choice of reflections for refinement. R-factors based on F^2^ are statistically about twice as large as those based on F, and R- factors based on ALL data will be even larger.

### Fractional atomic coordinates and isotropic or equivalent isotropic displacement parameters (Å^2^)

**Table:**

	*x*	*y*	*z*	*U*_iso_*/*U*_eq_	
Br1	0.34848 (8)	0.78522 (2)	0.59104 (2)	0.06364 (14)	
N1	−0.1456 (5)	0.72632 (14)	0.78238 (14)	0.0388 (5)	
N2	−0.2377 (6)	0.7831 (2)	0.91338 (17)	0.0548 (6)	
C6	−0.0182 (6)	0.72501 (17)	0.70949 (16)	0.0400 (6)	
H6A	−0.0563	0.6812	0.6699	0.048*	
C5	0.1666 (6)	0.78770 (17)	0.69349 (17)	0.0424 (5)	
C4	0.2211 (6)	0.8540 (2)	0.7532 (2)	0.0508 (7)	
H4A	0.3458	0.8977	0.7425	0.061*	
C3	0.0906 (6)	0.8540 (2)	0.8269 (2)	0.0506 (7)	
H3A	0.1271	0.8977	0.8668	0.061*	
C2	−0.1011 (6)	0.78778 (19)	0.84360 (16)	0.0412 (6)	
O1	0.3976 (5)	0.64368 (15)	0.92821 (11)	0.0543 (6)	
O2	0.5093 (4)	0.59455 (14)	0.80384 (11)	0.0464 (5)	
O3	−0.1981 (4)	0.44309 (18)	1.10571 (14)	0.0577 (6)	
O4	0.1873 (5)	0.46064 (19)	1.16336 (14)	0.0582 (6)	
C7	0.3666 (5)	0.59209 (17)	0.86815 (13)	0.0345 (5)	
C8	0.1501 (6)	0.52312 (18)	0.86922 (15)	0.0402 (5)	
H8A	0.2232	0.4648	0.8554	0.048*	
H8B	0.0267	0.5384	0.8257	0.048*	
C9	0.0036 (6)	0.5146 (2)	0.95136 (17)	0.0405 (6)	
H9A	−0.0472	0.5740	0.9706	0.049*	
H9B	−0.1529	0.4795	0.9427	0.049*	
C10	0.1701 (6)	0.46966 (19)	1.01720 (16)	0.0426 (6)	
H10A	0.2264	0.4115	0.9965	0.051*	
H10B	0.3239	0.5060	1.0266	0.051*	
C11	0.0308 (5)	0.45656 (16)	1.09925 (16)	0.0369 (5)	
H1N1	−0.270 (6)	0.681 (2)	0.7886 (19)	0.037 (7)*	
H1N2	−0.335 (8)	0.740 (3)	0.923 (2)	0.045 (9)*	
H2N2	−0.188 (8)	0.813 (2)	0.954 (2)	0.054 (10)*	
H1O4	0.132 (11)	0.454 (3)	1.202 (3)	0.077 (15)*	

### Atomic displacement parameters (Å^2^)

**Table:**

	*U*^11^	*U*^22^	*U*^33^	*U*^12^	*U*^13^	*U*^23^
Br1	0.0722 (2)	0.05631 (18)	0.0624 (2)	−0.00719 (18)	0.02248 (18)	0.00228 (16)
N1	0.0394 (12)	0.0371 (10)	0.0400 (11)	−0.0100 (11)	−0.0015 (9)	−0.0063 (8)
N2	0.0634 (16)	0.0631 (15)	0.0380 (12)	−0.0189 (14)	−0.0009 (11)	−0.0141 (14)
C6	0.0444 (15)	0.0366 (12)	0.0390 (12)	−0.0025 (12)	−0.0050 (11)	−0.0050 (10)
C5	0.0438 (14)	0.0356 (11)	0.0479 (13)	−0.0004 (14)	0.0004 (12)	0.0015 (11)
C4	0.0439 (16)	0.0413 (14)	0.067 (2)	−0.0096 (13)	0.0036 (14)	−0.0033 (13)
C3	0.0520 (18)	0.0437 (13)	0.0562 (17)	−0.0128 (14)	−0.0056 (14)	−0.0111 (13)
C2	0.0463 (15)	0.0392 (12)	0.0380 (12)	−0.0033 (12)	−0.0070 (11)	−0.0055 (11)
O1	0.0721 (16)	0.0581 (11)	0.0327 (9)	−0.0205 (12)	0.0154 (9)	−0.0153 (8)
O2	0.0526 (12)	0.0619 (11)	0.0247 (8)	−0.0177 (11)	0.0048 (8)	−0.0074 (8)
O3	0.0367 (11)	0.0894 (16)	0.0470 (12)	−0.0027 (11)	0.0051 (9)	0.0139 (11)
O4	0.0492 (13)	0.0935 (18)	0.0320 (10)	−0.0119 (13)	0.0026 (10)	0.0173 (11)
C7	0.0397 (13)	0.0409 (12)	0.0229 (10)	−0.0022 (12)	−0.0023 (10)	0.0006 (8)
C8	0.0458 (14)	0.0450 (13)	0.0299 (11)	−0.0065 (13)	−0.0016 (11)	0.0015 (10)
C9	0.0369 (14)	0.0496 (13)	0.0349 (12)	−0.0016 (12)	0.0020 (11)	0.0095 (11)
C10	0.0399 (14)	0.0534 (14)	0.0344 (12)	0.0064 (14)	0.0079 (11)	0.0096 (10)
C11	0.0432 (14)	0.0351 (11)	0.0324 (12)	0.0038 (10)	0.0077 (11)	0.0064 (10)

### Geometric parameters (Å, °)

**Table:**

Br1—C5	1.890 (3)	O2—C7	1.266 (3)
N1—C6	1.340 (4)	O3—C11	1.201 (4)
N1—C2	1.360 (3)	O4—C11	1.307 (4)
N1—H1N1	0.94 (3)	O4—H1O4	0.69 (5)
N2—C2	1.323 (4)	C7—C8	1.514 (4)
N2—H1N2	0.83 (4)	C8—C9	1.522 (4)
N2—H2N2	0.83 (4)	C8—H8A	0.9700
C6—C5	1.356 (4)	C8—H8B	0.9700
C6—H6A	0.9300	C9—C10	1.515 (4)
C5—C4	1.402 (4)	C9—H9A	0.9700
C4—C3	1.359 (5)	C9—H9B	0.9700
C4—H4A	0.9300	C10—C11	1.510 (4)
C3—C2	1.419 (4)	C10—H10A	0.9700
C3—H3A	0.9300	C10—H10B	0.9700
O1—C7	1.241 (3)		
			
C6—N1—C2	123.8 (2)	O1—C7—C8	120.3 (2)
C6—N1—H1N1	114.6 (19)	O2—C7—C8	117.1 (2)
C2—N1—H1N1	121.7 (19)	C7—C8—C9	115.5 (2)
C2—N2—H1N2	122 (2)	C7—C8—H8A	108.4
C2—N2—H2N2	119 (3)	C9—C8—H8A	108.4
H1N2—N2—H2N2	116 (4)	C7—C8—H8B	108.4
N1—C6—C5	119.9 (2)	C9—C8—H8B	108.4
N1—C6—H6A	120.0	H8A—C8—H8B	107.5
C5—C6—H6A	120.0	C10—C9—C8	111.0 (2)
C6—C5—C4	119.6 (3)	C10—C9—H9A	109.4
C6—C5—Br1	119.9 (2)	C8—C9—H9A	109.4
C4—C5—Br1	120.5 (2)	C10—C9—H9B	109.4
C3—C4—C5	119.6 (3)	C8—C9—H9B	109.4
C3—C4—H4A	120.2	H9A—C9—H9B	108.0
C5—C4—H4A	120.2	C11—C10—C9	113.2 (2)
C4—C3—C2	120.5 (3)	C11—C10—H10A	108.9
C4—C3—H3A	119.7	C9—C10—H10A	108.9
C2—C3—H3A	119.7	C11—C10—H10B	108.9
N2—C2—N1	119.0 (3)	C9—C10—H10B	108.9
N2—C2—C3	124.4 (3)	H10A—C10—H10B	107.7
N1—C2—C3	116.6 (3)	O3—C11—O4	123.1 (3)
C11—O4—H1O4	117 (5)	O3—C11—C10	124.3 (3)
O1—C7—O2	122.6 (3)	O4—C11—C10	112.7 (2)
			
C2—N1—C6—C5	−0.2 (4)	C4—C3—C2—N2	−179.7 (3)
N1—C6—C5—C4	−0.6 (4)	C4—C3—C2—N1	−0.4 (4)
N1—C6—C5—Br1	179.5 (2)	O1—C7—C8—C9	−7.5 (4)
C6—C5—C4—C3	0.8 (5)	O2—C7—C8—C9	173.0 (2)
Br1—C5—C4—C3	−179.2 (3)	C7—C8—C9—C10	−71.7 (3)
C5—C4—C3—C2	−0.3 (5)	C8—C9—C10—C11	−178.0 (2)
C6—N1—C2—N2	−179.9 (3)	C9—C10—C11—O3	32.0 (4)
C6—N1—C2—C3	0.7 (4)	C9—C10—C11—O4	−148.6 (3)

### Hydrogen-bond geometry (Å, °)

**Table:**

*D*—H···*A*	*D*—H	H···*A*	*D*···*A*	*D*—H···*A*
N1—H1N1···O2^i^	0.94 (3)	1.73 (3)	2.666 (3)	177 (2)
N2—H1N2···O1^i^	0.83 (4)	1.99 (4)	2.806 (4)	170 (4)
N2—H2N2···O1^ii^	0.83 (3)	2.04 (3)	2.848 (3)	164 (3)
O4—H1O4···O2^iii^	0.69 (5)	1.93 (5)	2.601 (3)	166 (5)
C3—H3A···O3^iv^	0.93	2.57	3.382 (4)	146
C6—H6A···O3^v^	0.93	2.46	3.337 (4)	157

Symmetry codes: (i) *x*−1, *y*, *z*; (ii) *x*−1/2, −*y*+3/2, −*z*+2; (iii) −*x*+1/2, −*y*+1, *z*+1/2; (iv) *x*+1/2, −*y*+3/2, −*z*+2; (v) −*x*−1/2, −*y*+1, *z*−1/2.
